# Dry Socket Prevalence and Risk Factors in Third Molar Extractions: A Prospective Observational Study

**DOI:** 10.7759/cureus.56721

**Published:** 2024-03-22

**Authors:** Parul Tandon, Sushil Kumar Sahoo, Liza Mohanty, Nupur Jain, Vidya Hittalamani, Swapnali Shinde Kamble, Ramanpal Singh

**Affiliations:** 1 Department of Oral and Maxillofacial Surgery, Saraswati Dental College & Research Centre, Lucknow, IND; 2 Department of Oral and Maxillofacial Surgery, Hi-Tech Dental College and Hospital, Bhubaneswar, IND; 3 Department of Dentistry, Government Medical College & Hospital, Sundargarh, IND; 4 Department of Oral and Maxillofacial Surgery, Rungta College of Dental Sciences & Research, Bhilai, IND; 5 Department of Prosthodontics and Crown and Bridge, Yogita Dental College, Khed, IND; 6 Department of Pedodontics and Preventive Dentistry, Child Dental Home, Mumbai, IND; 7 Department of Oral Medicine and Radiology, New Horizon Dental College & Research Institute, Bilaspur, IND

**Keywords:** postoperative pain, surgical technique, oral hygiene, smoking, oral surgery, risk factors, prevalence, third molar extraction, alveolar osteitis, dry socket

## Abstract

Background

Third molar extraction is a routine oral surgical procedure that is often complicated by the development of a dry socket (alveolar osteitis). This prospective observational study aimed to investigate the prevalence of dry sockets and identify associated risk factors and causes, contributing to a comprehensive understanding of the postoperative outcomes of oral surgery.

Methods

This study employed a prospective observational design with a 12-month follow-up period. Participants aged 18-40 years scheduled for third molar extraction were included, whereas those with coagulopathies, pregnant or lactating women, patients with vitamin deficiencies, and individuals on medications affecting healing were excluded. Data collection involved comprehensive assessments at baseline, intraoperative details, and postoperative evaluations at 48 hours, one week, and two weeks. Statistical analyses included descriptive statistics, chi-square tests, t-tests, or Mann-Whitney U tests, and logistic regression for the risk factor analysis.

Results

A total of 238 participants with diverse demographic characteristics were enrolled in this study. The prevalence of dry sockets increased progressively from 20.6% at 48 hours to 41.2% at two weeks post-extraction. Smoking, poor oral hygiene, and surgical technique emerged as significant risk factors, with corresponding odds ratios of 6.41 (95% CI: 2.86-14.36, p < 0.001), 9.53 (95% CI: 2.12-42.84, p = 0.003), and 3.27 (95% CI: 2.08-5.15, p < 0.001), respectively. Pain intensity, measured using a Visual Analog Scale, gradually decreased from 48 hours to two weeks post-extraction.

Conclusion

This study provides valuable insights into the prevalence and risk factors associated with dry sockets following third molar extractions. Smoking, poor oral hygiene, and poor surgical techniques were identified as significant contributors, emphasizing the importance of preoperative counseling and targeted interventions.

## Introduction

Third molar extraction is a routine oral surgical procedure performed to alleviate various dental issues, including impaction, crowding, and infection [1]. Despite the routine nature of these extractions, the occurrence of dry socket or alveolar osteitis poses a significant postoperative challenge [2]. Dry sockets are characterized by severe pain, inflammation, and delayed wound healing at the extraction site, leading to increased patient discomfort and potential complications [3].

The prevalence of dry sockets ranges from 1% to 5% in routine dental extractions to as high as 30% in the case of surgically extracted third molars, depending on factors such as surgical technique, patient demographics, and postoperative care [4]. The variability in the reported prevalence underscores the complexity of this postoperative complication and the need for a more comprehensive understanding of its determinants.

Several studies have explored the risk factors associated with dry sockets, revealing a multifactorial etiology. Smoking has consistently emerged as a significant risk factor, with nicotine’s vasoconstrictive effects compromising the blood supply to the healing socket [5]. Poor oral hygiene practices have also been implicated, emphasizing the importance of preoperative patient education and oral care [6]. Additionally, surgical factors, such as the complexity of the extraction and choice of surgical technique, may influence the likelihood of dry socket development [2,7].

While the existing literature provides valuable insights, gaps remain in our understanding of the prevalence and interplay of risk factors associated with dry socket following third molar extractions. This prospective observational study sought to address these gaps by systematically investigating the prevalence of dry sockets and identifying potential risk factors and causes.

The significance of this study lies in its potential to guide clinicians in identifying patients at a higher risk for dry socket, allowing for targeted preventive measures and postoperative care strategies. Understanding the prevalence and risk factors associated with this complication is crucial for optimizing patient care and reducing the overall burden of postoperative complications during oral surgery.

## Materials and methods

This was a prospective observational study spanning 12 months. The goal of this study was to systematically observe and document the occurrence of dry sockets in patients undergoing third molar extractions while identifying potential causative factors. Ethical considerations for this study involved obtaining approval from the Institutional Review Board of Saraswati Dental College and Research Center with the number IEC/SDC/2021/223 and securing written informed consent from all the participants.

Participants eligible for enrollment in the study were required to meet specific criteria, including being aged between 18 and 40 years and scheduled for third molar extraction. However, patients meeting any of the following criteria were excluded from the study: a history of coagulopathies; individuals with known bleeding disorders to minimize complications during and after the surgical procedure; pregnant or breastfeeding women due to potential impacts of the study procedures and radiation exposure; and patients currently taking medications known to affect wound healing (e.g., immunosuppressants and steroids) to control for confounding variables.

Comprehensive demographic information was collected from each participant at the outset of the study. This included details such as age, sex, and relevant aspects of medical history. The initial data were used to establish a foundation for understanding the characteristics of the study population. A thorough preoperative assessment was conducted, in addition to demographic information. This involved evaluating the participants’ oral hygiene practices and documenting their smoking status. Scaling was performed before surgery. These data points were deemed crucial for exploring potential associations between preexisting conditions and the development of dry sockets post-extraction. During the surgical procedure, detailed information regarding the specific technique employed for the extraction of the third molar was recorded. This included whether a simple extraction or a more complex surgical technique was used. The duration of each surgical procedure was meticulously documented. This parameter was used to capture any variations in surgical time and to assess whether prolonged interventions correlated with an increased risk of dry socket development.

Postoperative evaluation was performed at distinct time points, specifically 48 hours, one week, and two weeks after the extraction. The postoperative medications were prescribed (Tab Amoxicillin 500 mg and Tab Zerodol for five days). This phased approach allowed for a comprehensive examination of the postoperative period, capturing both the immediate and short-term outcomes. The identification of dry socket cases was performed using standardized criteria established a priori. These criteria encompassed clinical signs and symptoms such as severe pain and delayed wound healing. The systematic application of consistent diagnostic criteria ensured the reliability and comparability of the findings. To quantify and qualify postoperative pain experiences, a Visual Analog Scale (VAS) was employed. Participants were asked to rate their pain levels on a scale of 0-10, providing valuable insights into the intensity of pain experienced during the postoperative period [8].

Statistical analyses were performed using IBM SPSS Statistics for Windows, Version 23.0 (Released 2015; IBM Corp., Armonk, NY, USA). Descriptive statistics, including frequencies, percentages, means, and standard deviations, were used to characterize the study population and provide a detailed overview of the key variables. For inferential analysis, chi-square tests were employed to explore associations between categorical variables, whereas t-tests or Mann-Whitney U tests were used for continuous variables. These inferential tests aimed to identify the potential correlations between various factors and the occurrence of dry sockets. Furthermore, logistic regression analysis was employed to delve deeper into the identification of risk factors, allowing for a nuanced understanding of the predictors contributing to the development of dry sockets post-extraction. The significance level for all tests was defined as a p-value ≤ 0.05.

## Results

The study enrolled 238 participants, with a notable distribution across age groups. The majority were in the 18-25 (38.7%) and 26-30 (31.1%) age brackets, ensuring diverse representation. The sex distribution was nearly equal, with 48.7% males and 51.3% females (Table 1).

**Table 1:** Demographic characteristics of study participants

Characteristic	Category	Frequency (n = 238)	Percentage
Age (years)	18-25	92	38.70%
	26-30	74	31.10%
	31-40	72	30.20%
Gender	Male	116	48.70%
	Female	122	51.30%

The prevalence of dry sockets progressively increased during the postoperative period. At 48 hours, 20.6% of participants experienced a dry socket, which rose to 31.9% at one week and peaked at 41.2% at the two-week mark. These differences were statistically significant (p < 0.05), indicating a substantial incidence of delayed healing, with the highest prevalence observed around two weeks post-extraction (Table 2).

**Table 2:** Prevalence of dry sockets at different time points

Time point	No dry socket (n)	Dry socket (n)	Prevalence (%)
Forty-eight hours	189	49	20.60%
One week	162	76	31.90%
Two weeks	140	98	41.20%

Smoking and poor oral hygiene were identified as substantial risk factors for the development of dry sockets. Participants who smoked had an odds ratio of 6.41 (95% CI: 2.86-14.36, p < 0.001), while those with poor oral hygiene had an odds ratio of 9.53 (95% CI: 2.12-42.84, p = 0.003). Additionally, the choice of surgical technique was significant, with surgical extractions having an odds ratio of 3.27 (95% CI: 2.08-5.15, p < 0.001) compared with simple extractions (Table 3).

**Table 3:** Risk factors associated with dry socket p-value was considered significant if ≤0.05.

Variable	Dry socket (n = 223)	No dry socket (n = 15)	Odds ratio (95% CI)	p-value
Smoking	132	8	6.41 (2.86-14.36)	<0.001
Poor oral hygiene	95	2	9.53 (2.12-42.84)	0.003
Surgical technique				
Simple extraction	45	67	Reference	-
Surgical extraction	178	82	3.27 (2.08-5.15)	<0.001

The participants reported varying pain intensity scores over time. At 48 hours, the mean pain score was 6.8 (SD 2.1), which decreased to 4.2 (SD 1.5) by one week and further reduced to 2.1 (SD 1.0) by two weeks. These differences were statistically significant (p < 0.05), indicating a consistent decline in postoperative pain, which was consistent with the expected healing trajectory (Table 4).

**Table 4:** Pain intensity scores (VAS) at 48 hours, one week, and two weeks VAS, Visual Analog Scale

Time point	Mean pain score (SD)
Forty-eight hours	6.8 (2.1)
One week	4.2 (1.5)
Two weeks	2.1 (1.0)

## Discussion

The findings of this prospective observational study shed light on the prevalence and associated factors of dry socket following third molar extraction. This comprehensive analysis encompassed demographic characteristics, risk factors, and postoperative pain experiences, providing valuable insights for clinicians and researchers in the field of oral surgery. The study observed an increasing prevalence of dry sockets over the postoperative period, with statistically significant differences noted at each assessment time point (p < 0.05). This aligns with the existing literature indicating that dry socket is a common complication following third molar extractions, with peak occurrences typically observed in the first week post-surgery [9]. The observed prevalence rates, reaching 41.2% at the two-week mark, emphasize the importance of understanding and managing this complication to enhance patient outcomes.

Smoking and poor oral hygiene have emerged as significant risk factors for the development of dry sockets. The odds ratios for these factors were notably high, emphasizing their clinical relevance. The association between smoking and an increased risk of dry sockets is well documented [10,11]. The vasoconstrictive effects of nicotine can compromise the blood supply to the surgical site, delay healing, and increase the likelihood of dry socket development [12,13]. Additionally, poor oral hygiene has been consistently linked to postoperative complications [4,14]. Inadequate oral hygiene may introduce bacteria into the extraction site, leading to inflammation and delayed wound healing [15]. The observed association with the choice of surgical technique is noteworthy, as complex surgical extractions demonstrated higher odds of dry sockets than simple extractions. This underscores the importance of careful consideration when choosing an appropriate surgical approach with a potential impact on postoperative outcomes [7].

The VAS, which measures pain intensity scores, showed a consistent decline over the postoperative period. The statistically significant differences at each time point (p < 0.05) reflected the expected trajectory of postoperative pain following third molar extractions. The initial high pain scores at 48 hours gradually diminished, reaching a mean score of 2.1 at two weeks. This aligns with studies emphasizing that postoperative pain tends to peak within the first 48 hours and subsequently decreases as healing progresses [16,17]. The observed pattern underscores the importance of effective pain management during the immediate postoperative period, with subsequent improvements in patient comfort over time.

Effective pain management is crucial not only for patient comfort but also for promoting optimal wound healing [18]. The gradual decrease in pain scores over time suggests a positive healing trajectory, with patients experiencing a reduction in discomfort as the extraction site undergoes expected reparative processes. This finding is consistent with the existing literature on postoperative pain following third molar extractions [19,20]. The study done by Murthi et al. found that the prevalence of dry sockets was 4.05%, and 95.95% of the patients did not experience dry sockets [21]. The dry socket remains the most common postoperative complication. The dry socket was most commonly associated with males and during normal extraction (i.e., without surgical exposure of the flap and tooth). The identification of smoking, poor oral hygiene, and the choice of surgical technique as significant contributors to dry socket development have immediate clinical implications. Clinicians should integrate these findings into their preoperative assessments, risk stratification, and postoperative care plans. Tailored interventions, such as intensified postoperative care for patients with identified risk factors, may contribute to reducing the incidence of dry sockets and enhancing overall patient outcomes. The clinical recommendations stemming from this study align with the best established practices in oral surgery. Smoking cessation programs, preoperative oral hygiene counseling, and careful consideration of surgical approaches are essential components of patient care. Additionally, the findings underscore the importance of comprehensive postoperative monitoring, with a focus on the critical 48 hours to two weeks post-extraction.

The strengths of this study lie in its prospective design, large sample size, and detailed data collection, which enhanced the reliability and applicability of the findings. The use of standardized criteria for dry socket diagnosis further strengthened the study’s internal validity. However, similar to any other research endeavor, there are limitations to consider. The age range restriction (18-40 years) may limit the generalizability of our findings to older or younger populations. Although efforts were made to control for confounding variables, unmeasured factors may still contribute to the observed outcomes. The focus of this study on a specific demographic should be acknowledged when applying these findings to a broader patient population.

Future research in this area could explore additional risk factors, such as systemic health conditions and genetic predispositions, that contribute to dry socket development. Long-term follow-up studies extending beyond the two-week period could provide insights into the extended healing process and potential delayed complications. Comparative studies assessing the efficacy of different postoperative care interventions may contribute to refining the clinical guidelines for third molar extraction.

## Conclusions

This prospective observational study provides valuable insights into the prevalence and causes of dry sockets following third molar extraction. The identified risk factors, including smoking, poor oral hygiene, and choice of surgical technique, underscore the multifactorial nature of this complication. These findings contribute to the evidence-based guidance for clinicians in risk assessment and postoperative care. Ongoing research and collaboration within the dental and surgical communities will further refine our understanding and ultimately improve patient outcomes.

## References

[1] Muhsin H, Brizuela M (2024). Oral surgery, extraction of mandibular third molars. StatPearls [Internet].

[2] Mamoun J (2018). Dry socket etiology, diagnosis, and clinical treatment techniques. J Korean Assoc Oral Maxillofac Surg.

[3] Akinbami BO, Godspower T (2023). Dry socket. Int J Dent.

[4] Daly BJ, Sharif MO, Jones K, Worthington HV, Beattie A (2022). Local interventions for the management of alveolar osteitis (dry socket). Cochrane Database Syst Rev.

[5] Albanese M, Zangani A, Manfrin F (2023). Influence of surgical technique on post-operative complications in the extraction of the lower third molar: a retrospective study. Dent J (Basel).

[6] Akram A (2023). Literature review of dry socket: etiology, pathogenesis, prevention, and management. Int J Community Med Public Health.

[7] Taberner-Vallverdú M, Sánchez-Garcés MÁ, Gay-Escoda C (2017). Efficacy of different methods used for dry socket prevention and risk factor analysis: a systematic review. Med Oral Patol Oral Cir Bucal.

[8] Jensen MP, Chen C, Brugger AM (2003). Interpretation of visual analog scale ratings and change scores: a reanalysis of two clinical trials of postoperative pain. J Pain.

[9] Abu Younis MH, Abu Hantash RO (2011). Dry socket: frequency, clinical picture, and risk factors in a palestinian dental teaching center. Open Dent J.

[10] Kuśnierek W, Brzezińska K, Nijakowski K, Surdacka A (2022). Smoking as a risk factor for dry socket: a systematic review. Dent J (Basel).

[11] Majid OW (2023). Further evidence confirms the association between smoking and dry socket: a motivational opportunity for tobacco cessation. Evid Based Dent.

[12] Sanari AA, Alsolami BA, Abdel-Alim HM, Al-Ghamdi MY, Meisha DE (2020). Effect of smoking on patient-reported postoperative complications following minor oral surgical procedures. Saudi Dent J.

[13] Malhotra R, Kapoor A, Grover V, Kaushal S (2010). Nicotine and periodontal tissues. J Indian Soc Periodontol.

[14] Taberner-Vallverdú M, Camps-Font O, Gay-Escoda C, Sánchez-Garcés MA (2022). Previous dry socket as a risk factor for alveolar osteitis: a nested case-control study in primary healthcare services. J Clin Exp Dent.

[15] Rohe C, Schlam M (2024). Alveolar osteitis. StatPearls [Internet].

[16] de Santana-Santos T, de Souza-Santos aA, Martins-Filho PR, da Silva LC, de Oliveira E Silva ED, Gomes AC (2013). Prediction of postoperative facial swelling, pain and trismus following third molar surgery based on preoperative variables. Med Oral Patol Oral Cir Bucal.

[17] de Santana Santos T, Calazans AC, Martins-Filho PR, Silva LC, de Oliveira E Silva ED, Gomes AC (2011). Evaluation of the muscle relaxant cyclobenzaprine after third-molar extraction. J Am Dent Assoc.

[18] Bechert K, Abraham SE (2009). Pain management and wound care. J Am Col Certif Wound Spec.

[19] Avellaneda-Gimeno V, Figueiredo R, Valmaseda-Castellón E (2017). Quality of life after upper third molar removal: a prospective longitudinal study. Med Oral Patol Oral Cir Bucal.

[20] Bortoluzzi MC, Guollo A, Capella DL (2011). Pain levels after third molar surgical removal: an evaluation of predictive variables. J Contemp Dent Pract.

[21] Murthi M, Dhasarathan P, Rajendran D (2020). Retrospective study of the prevalence of dry socket in patients with mandibular third molar extraction. World J Dent.

